# Performance Assessment of an Electrostatic Filter-Diverter Stent Cerebrovascular Protection Device: Evaluation of a Range of Potential Electrostatic Fields Focusing on Small Particles

**DOI:** 10.3390/bioengineering11111127

**Published:** 2024-11-08

**Authors:** Beatriz Eguzkitza, José A. Navia, Guillaume Houzeaux, Constantine Butakoff, Mariano Vázquez

**Affiliations:** 1 Barcelona Supercomputing Center, Computer Applications in Science and Engineering, 08034 Barcelona, Spain; guillaume.houzeaux@bsc.es (G.H.); mariano@elem.bio (M.V.); 2 Cardiac Surgery Service Department, University Hospital, Austral University, Pilar B1630FHB, Buenos Aires, Argentina; jose.navia@yahoo.com; 3 ELEM Biotech SL, 08003 Barcelona, Spain; cbutakoff@elem.bio

**Keywords:** computational fluid dynamics modeling (CFD), electrical repulsion, atrial fibrillation, TAVR, silent brain infarcts, stroke, particle flow simulation, cerebroembolic protection devices, aortic arch

## Abstract

Silent Brain Infarction (SBI) is increasingly recognized in patients with cardiac conditions, particularly Atrial Fibrillation (AF) in elderly patients and those undergoing Transcatheter Aortic Valve Implantation (TAVI). While these infarcts often go unnoticed due to a lack of acute symptoms, they are associated with a threefold increase in stroke risk and are considered a precursor to ischemic stroke. Moreover, accumulating evidence suggests that SBI may contribute to the development of dementia, depression, and cognitive decline, particularly in the elderly population. The burden of SBI is substantial, with studies showing that up to 11 million Americans may experience a silent stroke annually. In AF patients, silent brain infarcts are common and can lead to progressive brain damage, even in those receiving anticoagulation therapy. The use of cerebral embolic protection devices (CEPDs) during TAVI has been explored to mitigate the risk of stroke; however, their efficacy remains under debate. Despite advancements in TAVI technology, cerebrovascular events, including silent brain lesions, continue to pose significant challenges, underscoring the need for improved preventive strategies and therapeutic approaches. We propose a device consisting of a strut structure placed at the base of the treated artery to model the potential risk of cerebral embolisms caused by atrial fibrillation, thromboembolism, or dislodged debris of varying potential TAVI patients. The study has been carried out in two stages. Both are based on computational fluid dynamics (CFD) coupled with the Lagrangian particle tracking method. The first stage of the work evaluates a variety of strut thicknesses and inter-strut spacings, contrasting with the device-free baseline geometry. The analysis is carried out by imposing flow rate waveforms characteristic of healthy and AF patients. Boundary conditions are calibrated to reproduce physiological flow rates and pressures in a patient’s aortic arch. In the second stage, the optimal geometric design from the first stage was employed, with the addition of lateral struts to prevent the filtration of particles and electronegatively charged strut surfaces, studying the effect of electrical forces on the clots if they are considered charged. Flowrate boundary conditions were used to emulate both healthy and AF conditions. Results from numerical simulations coming from the first stage indicate that the device blocks particles of sizes larger than the inter-strut spacing. It was found that lateral strut space had the highest impact on efficacy. Based on the results of the second stage, deploying the electronegatively charged device in all three aortic arch arteries, the number of particles entering these arteries was reduced on average by 62.6% and 51.2%, for the healthy and diseased models respectively, matching or surpassing current oral anticoagulant efficacy. In conclusion, the device demonstrated a two-fold mechanism for filtering emboli: (1) while the smallest particles are deflected by electrostatic repulsion, avoiding micro embolisms, which could lead to cognitive impairment, the largest ones are mechanically filtered since they cannot fit in between the struts, effectively blocking the full range of particle sizes analyzed in this study. The device presented in this manuscript offers an anticoagulant-free method to prevent stroke and SBIs, imperative given the growing population of AF and elderly patients.

## 1. Introduction

Is it possible to mitigating elderly patients’ SBI during (AF) and transcatheter aortic valve implantation TAVI? The object of this paper is to demonstrate an affirmative answer thanks to an innovative device.

SBI is increasingly observed in patients with cardiac disease and in individuals who have undergone invasive cardiac procedures. Cardiac diseases associated with the occurrence of SBIs include AF, cardiomyopathies, and atrial septal abnormalities. Postprocedural SBIs have been detected using MRI after left cardiac catheterization, TAVI, CABG surgery, pulmonary vein isolation (AF Ablation), and closure of patent foramen ovale. SBIs do not always result in acute symptoms, but have been associated with a three-fold increase in the risk of stroke, and can be considered a precursor of ischemic stroke. Accumulating evidence suggests that SBIs might have a role in the development of dementia, depression, and cognitive decline. Increased recognition of SBIs could advance our understanding of their links to cardiac and neurological disorders, and facilitate the development of a prevented therapeutics approach [1]. Cerebral small vessel disease (SVD) refers to the presence of brain lesions, found on CT or MR brain imaging or pathology examination, thought to have resulted from disease of the small blood vessels that perforate into the brain, primarily the white matter and deep grey matter. The best-described pathological-anatomical cerebral lesions are white matter hyperintensities (WMH), lacunes, microbleeds, and enlarged perivascular spaces. SVD is considered to cause 25% of ischemic strokes and most hemorrhagic strokes in older patients, is also the most common cause of vascular cognitive impairment and vascular dementia and is common in mixed dementia with other dementia pathologies [2,3]. Cerebral SVD has several clinical and radiographic manifestations, lacunar stroke (LS) is prototypical and for 20–30% of ischemic strokes affects a range of vessel sizes, from 40–900 µm in diameter [4]. Cerebral SVD is considered to cause 25% of ischaemic strokes and most hemorrhagic strokes in older patients [3] causing the majority of silent cerebral infarctions. Micro-embolisms from atrial fibrillation (AF) and those confirmed by magnetic resonance imaging (MRI) of aortic valve prosthetic implants (TAVI) (60–90% of silent infarctions [5] could theoretically be positively influenced by reducing one of the causes of SBI, benefiting the reduction of cognitive decline and subsequent progression to dementia in these patients.

Compared with symptomatic cerebral infarction, the lesions of SBI are usually relatively smaller in size and may have undergone a chronic ischemic preconditioning process, contributing to the absence of clinical symptoms. Previous reports have established that SBI is prevalent in both healthy older adults as well as in specific populations, such as those with hypertension, diabetes, atrial fibrillation, and other conditions. SBI with an infarct diameter greater than 3mm, were defined as hypointense lesions on T1WI, while on dT2WI they were characterized as hyperdense [6]. The global burden of stroke and dementia is increasing. If current trends continue, by 2050 we can expect about 200 million stroke survivors 106 million people with dementia, and each year thereafter, over 30 million new strokes, 12 million deaths from stroke, and almost 5 million deaths from dementia. This looming future will threaten the sustainability of health systems worldwide [7]. Yet it is preventable, as a substantial proportion of the burden is attributable to risk factors that can be modified. Thus, the timely and accurate prognosis of stroke outcomes based on clinical or radiological markers is vital for both physicians and stroke survivors. Among radiological markers, cerebral microbleeds (CMBs) constitute markers of blood leakage from pathologically fragile small vessels. The utility of CMB assessments, not only in the prognostication of hemorrhagic complications of reperfusion therapy, but also in forecasting hemorrhagic and ischemic stroke patients’ functional outcomes, thus indicating that a biomarker-based approach may aid in, improving the selection of more appropriate medical therapies, and contribute to a more accurate choice of patients for reperfusion therapy. The radiological observation of tiny perivascular hemorrhages, usually seen in elderly individuals, appearing as hypointense, rounded lesions (2–10 mm in diameter), and related MRI sequences that are sensitive to magnetic susceptibility [8]. As far as the epidemiological profile of CMBs is concerned, age appears to be the most decisive risk factor, since their prevalence increases and a striking exacerbation after the age of 75 has been observed. Additionally, male sex, systemic hypertension, and heavy smoking have been linked with CMB development [9]. Since the presence of CMBs denotes the occurrence of blood leakage from pathologically fragile small vessels, a fundamental question may be raised regarding whether CMBs may shift the risk-benefit balance away from antithrombotic use in some patients. Another relevant clinical dilemma is whether CMBs increase the risk of intracerebral hemorrhage (ICH) after intravenous thrombolysis (IVT) or another type of reperfusion therapy for patients with acute ischemic stroke (AIS). Taking into consideration the clinical relevance and the potential prognostic role of CMBs within an aging population, as well as the emerging need for accurate forecasting of each stroke individual’s propensity for recovery, the purpose of the present study was to review all available literature published within the last decade dealing with CMBs as outcome predictors not only after reperfusion therapy for AIS but also in hemorrhagic and AIS survivors who had not undergone reperfusion therapy [7,10].

SBI and Atrial FibrillationAtrial fibrillation (AF) is a common type of arrhythmia. There are currently 335 million individuals with AF worldwide, with an overall prevalence rate of 2.9% With an aging global population and changing lifestyles, the incidence of AF is increasing rapidly. The prevalence of AF is around 0.1% for individuals under 55 years old, more than 5% for people over 65 years old, and more than 9% for people over 80 years old [11,12]. AF has been shown to increase the risk of dementia progression in patients and has been correlated with decreased cognitive assessment scores over time. Several hypotheses have been proposed with varying levels of experimental evidence, and include macro- or microvascular stroke events, biochemical changes to the blood-brain barrier related to anticoagulation, or hypo- or hyperperfusion events identified an increased risk of cognitive decline of approximately 40–60%, these patients describe a mental ‘fog’ or lack of mental sharpness, due to significant hemodynamic stress on the central nervous system (CNS), which can be detected via changes in brain perfusion. These effects are caused by beat-to-beat variability. an irregular heart rhythm, which develops subsequent impaired diastolic filling, acute and chronic stress responses, and maladaptive consequences that lead to vascular dysfunction [13]. As many as 11 million Americans may experience a silent stroke each year, whereas 800,000 have typical strokes with symptoms. According to the American Heart Association (AHA) and American Stroke Society, one in every four adults older than 80 years has had one or more of these strokes. The brain damage caused by such asymptomatic strokes is cumulative. A silent or asymptomatic stroke increases the chance of symptomatic brain strokes later in life (multi-infarct dementia) and paralysis A previous study identified risk factors for clinically symptomatic thromboembolism in patients with AF, including left atrial (LA) abnormalities (e.g., LA thrombus, spontaneous echo contrast [SEC], abnormal LA appendage emptying velocity (LAAeV]), and aortic abnormalities. Moreover, recent studies have suggested that, in AF patients, microembolization of small thrombi from the fibrillating appendage (LAA) plays an important role in the occurrence of SBI [2]. These consequences of stroke could increase the risks of death and disability by more than 5-fold. In general, the fatality rates for stroke are 15, 25, and 50% in the 1-month, 1-year, and 5-year post-stroke periods, respectively. Therefore, clinical guidelines have identified anticoagulation for individuals with NVAF(Nonvalvular AF), as the cornerstone approach to controlling ischemic stroke. However, since clinical risks of atrial fibrillation increase with age, more proactive prevention methods are needed for older individuals. In the Swiss-AF cohort, 5.5% of AF patients developed new brain infarcts after 2 years of follow-up. The great majority (85%) of these infarcts were clinically silent and occurred in anticoagulated patients. These data suggest that anticoagulation alone may not be sufficient to prevent progressive brain damage in all AF patients Ischemic brain infarcts were quite common in the unselected AF population over 2 years of follow-up and the great majority (85%) were clinically silent. About one in five patients with AF had clinically silent brain infarcts on systematic brain magnetic resonance imaging (MRI) [14]. Some reflections suggest that cardio-embolism is not the only possible explanation of the link between atrial arrhythmias and dementia.

In short: the temporal relationship between atrial fibrillation and ischemic stroke is often non-existent and today it is thought that the atrium itself can provide an embolic-prone environment even in the absence of arrhythmia (so-called atrial cardiomyopathy) [15].

SBI and TAVI (Trans aortic valve implantation) Despite the advancements and introduction of new-generation TAVI devices, cerebrovascular events (CVE) remain one of the most serious complications. Interestingly, the incidence of clinical CVE post-TAVI is less than 5%, and the incidence of silent lesions evaluated by diffusion-weighted MRI (DW-MRI) is 60–90%, irrespective of the type of device or access site. These silent lesions are associated with an increased risk of stroke, dementia, and long-term cognitive decline as well. A study conducted by Lansky et al. demonstrated that 94% of patients who underwent DW-MRI had brain lesions and 41% showed a cognitive decline from baseline to 30-day follow-up when assessed using Montreal Cognitive Assessment [5]. Recent trial data have led to the expansion of TAVI into lower-risk patients. With iterative technological advances and successive increases in procedural experience, the occurrence of complications following TAVI has declined. One of the most feared complications remains stroke, and patients consider stroke a worse outcome than death. There has therefore been great interest in strategies to mitigate the risk of stroke in patients undergoing TAVI. CEPDs (Cerebral Embolic Protection Devices) are deployed during the TAVI procedure to protect against embolism to the brain and consequent stroke and can be broadly classified into filters or deflectors. Filters achieve cerebral protection by capturing and extracting emboli from the circulation while Deflectors alternate the route of the emboli away from the cerebral circulation to the systemic circulation. The actual efficacy of the device depends on the capacity to protect the 3 main branches of the aortic arch. Filter size, and the ability to deploy without disrupting aortic arch plaque (which can itself lead to a risk of atheroembolism and stroke) [16]. There is a discrepancy between clinically apparent strokes and the incidence of new ischaemic lesions seen on MRI post-TAVI, suggesting that some lesions might be silent. These lesions may affect long-term cognitive status, as studies have shown that silent lesions may cause a more pronounced cognitive decline and an increased risk of dementia. This meta-analysis did not show a significant reduction in total new lesion volume, number of new lesions, or patients with new lesions on neuroimaging. The reasons for these findings could be multifactorial, as cerebral embolization during TAVI is still possible even with the SENTINEL device. First, the SENTINEL CPS only has filters covering the brachiocephalic trunk and left common carotid artery, thus, leaving the left vertebral artery unprotected and susceptible to embolism. Second, manipulation of the SENTINEL device within atherosclerotic arteries may cause embolism directly. Thrombi may also form on the surface of the protection device itself during the earlier phases of the procedure when full anticoagulation may not yet be in effect. Third, the SENTINEL filter only has 1 size, and malposition may occur, leading to incomplete sealing of arteries and allowing embolic debris to slip by. Fourth, embolic debris may be smaller than the pore size of the filters (140 µm in diameter), thus, it can pass through the filter. Lastly, the studies included in this meta-analysis also had several issues including incomplete MRI follow-ups and small numbers [17]. The Sentinel CEP can functionally capture large debris that may cause severe stroke. Most captured debris had a size of less than 500 µm (78% were 150–500 µm). Nearly 5% of the captured particles were greater or equal than 1000 µm, and these were detected in 67% of cases. These silent lesions are associated with an increased risk of stroke, dementia, and long-term cognitive decline as well. Despite the advancements and introduction of new-generation TAVI devices, cerebrovascular events (CVE) remain among the most serious complications [18].

## 2. Objectives and Methods

### 2.1. Objectives

This work aims to prevent the highest number of cardioembolism microparticles (10–600 µm) that cause asymptomatic multiple small brain infarcts (SBI). The theoretical study, through computational analyses, aims to demonstrate the efficiency, ϵ of the filter/diverter stent device, defined as

(1) $$\epsilon =(1-N_{f}/N_{nf})\times 100$$

where $N_{fq}$ is the number of particles passing through the selected artery with device (charged or neutral) and $N_{fn}$ represents the number of particles passing through the same artery without any filter. The prototype of the device, developed by Admedes Germany, consists of a self-expanding nitinol (cadmium-nickel) stent, coated with graphene oxide, plus bovine serum albumin or/and gold magnetic nanoparticles (MNAu), with a negative charge within physiological limits. This provides antifouling, antithrombotic, and antihemolytic properties by repelling blood cells and proteins, all negatively charged. The device will be implanted at the entrance of the supra-aortic trunks via catheter through arterial pathways [19,20].

The work presented in this paper represents an evolution of prior research and technological developments, particularly those protected by patents referenced as 10.470903, 9636.204, 10.695.199. These patents laid the foundation for the current advancements by addressing the initial design for our proposed filter device.

In this study, we build on these foundational innovations by introducing new technologies that enhance the deflection of smallest particles to avoid micro embolisms and address limitations present in earlier designs. The improvements introduced in this work—such as electrostatic repulsion forces expand the applicability of the original inventions and provide a more robust solution to prevent stroke and SBIs. This evolution reflects the natural progression of technology in this field, offering novel insights and capabilities while maintaining continuity with the earlier patented innovations.

### 2.2. Aortic Arch Geometry

**Figure 1:**
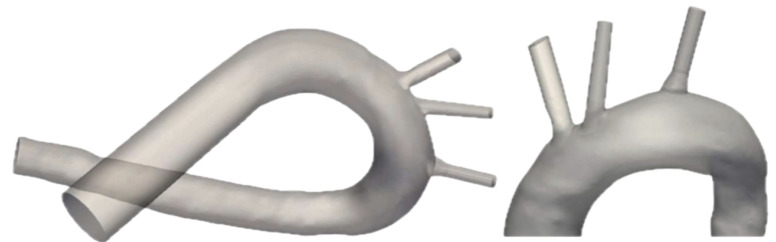
Aortic arch geometry. In the original geometry, the main vessels were segmented manually and recreated based on a CT scan of a cadaver. Left ventricle, aortic valve and coronary arteries have been replaced in the inlet by a rigid tube, and superior mesenteric, iliac and renal arteries have been dismissed at the outlet. As highlighted in the zoomed-in figure on the right, extrusion extensions have been employed at the vessel outlets to enhance the stability of the simulated flux.

The aortic arch geometry used for computational simulations was designed based on an anatomical model from a previous study [21]. The resulting geometry used in this study can be observed in Figure 1 and has been widely described in [22]. As it was described in detail in that reference, the aortic arch geometry for the simulations was adapted from a previous study’s anatomical model. To meet the current study’s needs. These changes maintained the overall structure, but significant alterations were applied to the LCCA and BCT, converting them from anatomical to synthetic forms. The BCT was manually adjusted to better reflect an average geometry, and the LCCA was corrected to avoid potential errors caused by its broader entrance. The aortic root was truncated to focus on the arch vessels, and the inlet was replaced by a rigid tube to stabilize fluid flow. Outlets to the superior mesenteric, iliac, and renal arteries were excluded.

**Figure 2:**
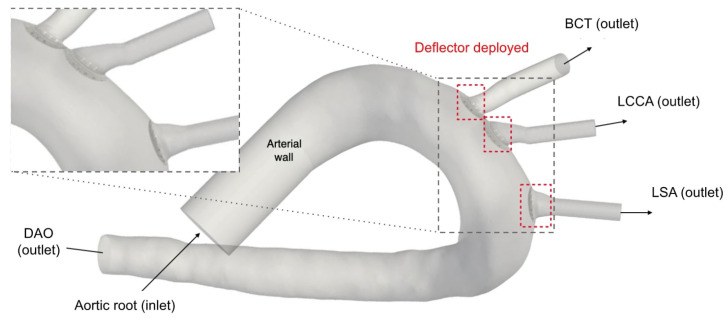
Geometry of the aortic arch and zoom-in of the deflectors deployed at all three arteries.

For the third stage of the present work, the device is implanted at the base of the three arteries: BCT, LCCA, and LSA. Figure 2 shows the patient-specific geometry of the aortic arch employed, with the arteries where the device will be placed. The inlet and outlet boundaries are labelled as:

- Inlet: Aortic root
- Outlets:
  − Brachiocephalic Trunk (BCT),
  − Left Carotid Common Artery (LCCA),
  − Left Subclavian Artery (LSA), and
  − Descending Aorta (DAO).

### 2.3. Device Design

#### Third Stage

**Figure 3:**
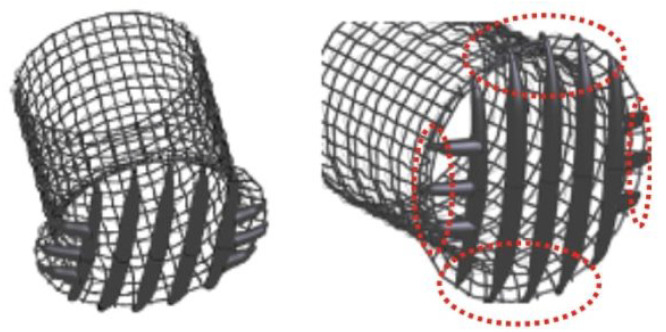
New deflector design. Lateral struts added are circled in red.

Extra struts has been added in the new design employed in the second stage of the present work, oriented perpendicularly to the original struts, as it is shown in Figure 3.

Figure 3 shows the new struts added to the device to improve the particle filtering in the previous stage of the present work [22]. Electric charge is also applied to additionally deflect small particles that could normally pass between the struts. The device would be covered with a negatively charged graphene oxide coating, with surface charges of −24,000 [statC ${\mathrm{cm}}^{2}$], which corresponds to the most extreme electrical charges found in the literature of biomedical devices, as was described in the supplemented material of [22]. StatC is a unit of electric charge in the centimeter-gram-second electrostatic system of units. One statCoulomb is the amount of charge that, if placed one centimeter apart from an equal charge in a vacuum, would repel it with a force of one dyne. To explore the capabilities of the electric field the present work evaluate different potential values that the device could offer, all of them inside the save regime as it has been studied in [23]. Four fields has been simulated as a results of a superficial charge in the device of the following values: −24,000, −48,000, −75,000, −100,000 [statC/m^2^] These values are below than the one described in the cited paper as the healthy limit in which cell damage occurs: 37.5 µm/cm^2^ which corresponds to a 112,275.45 [statC/m^2^].

## 3. Results

This section presents the efficacy obtained in this third stage of the work. As was concluded in the previous stages, the device has a two-fold deflecting/filtering mechanism: while the smallest particles are deflected by electrostatic repulsion (less than 50%), the largest ones are mechanically filtered since their diameter is similar or larger than the inter-strut spacing (100%). In consequence, the device effectively avoids a wide range of particle sizes from entering the aortic arch arteries, protecting the cerebrovascular system. In this new study, we are focused in the electrostatic repulsion for the smallest sizes. To that end, 4 different fields has been evaluated in 8 sizes from 10 to 600 µm. As we did in our previous stages, two different scenarios has been: healthy patient with TAVI and atrial fibrillation condition patient. It is important to highlight the efficiency of the microparticle rejection diverter effect In field 4, Atrial Fibrillation patients, with the maximum physiologic negative charge, it is possible to obtain positive results ranging from BCT (particles 10–500 microns) 100% to 40%, LCCA (particles 10–600 microns) 100% to 50%, LSA (particles 10–500 microns) 100% to 50% efficiency.

### 3.1. Healthy Patient(HP)/TAVI

#### Comparison Between 4 Intensities of Electric Field Repulsion and No-Charged Device

**Figure 4:**
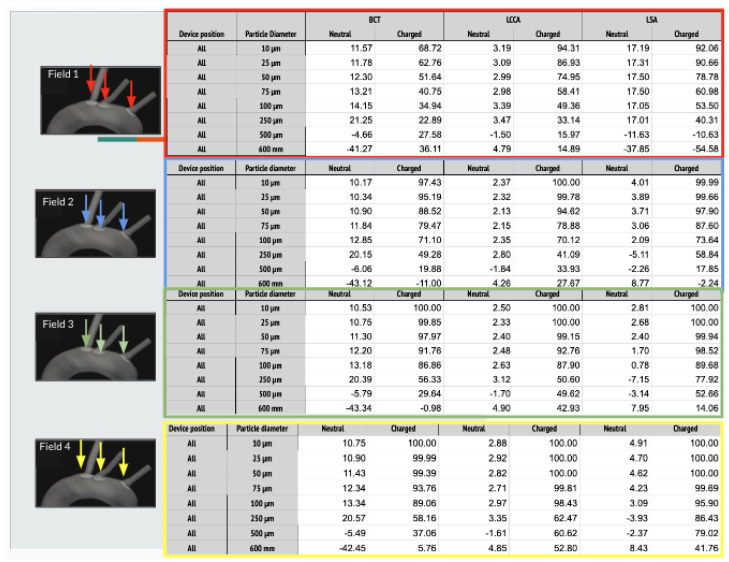
HP/TAVI: Tables of efficiency defined in Equation (1) for all electrical fields (ranging from 1 to 4 different intensities) generated with the applied superficial charge: (−24,000, −48,000, −75,000, −100,000 [statC/m^2^]) on the three devices positioned at the bases of the arteries, illustrated with the arrows.

**Figure 5:**
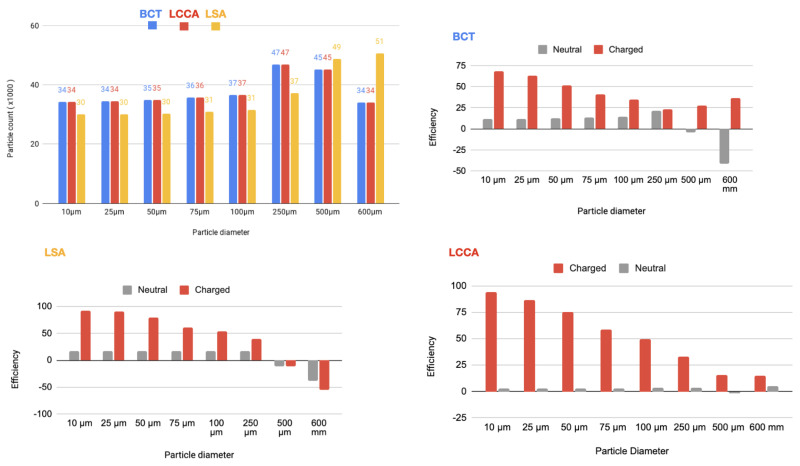
HP/TAVI—Field 1 (based on a device superficial charge of −24,000 statC/m^2^): Efficiency in each artery.

**Figure 6:**
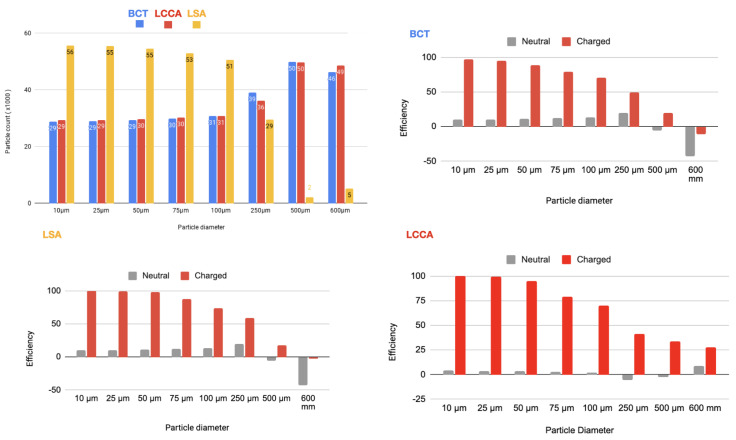
HP/TAVI—Field 2 (based on a device superficial charge of −48,000 [statC/m^2^]): Efficiency in each artery.

**Figure 7:**
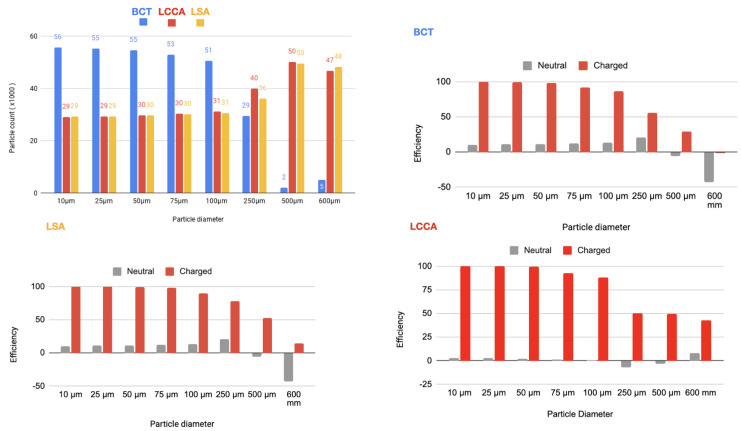
HP/TAVI—Field 3 (based on a device superficial charge of −75,000 [statC/m^2^]): Efficiency in each artery.

**Figure 8:**
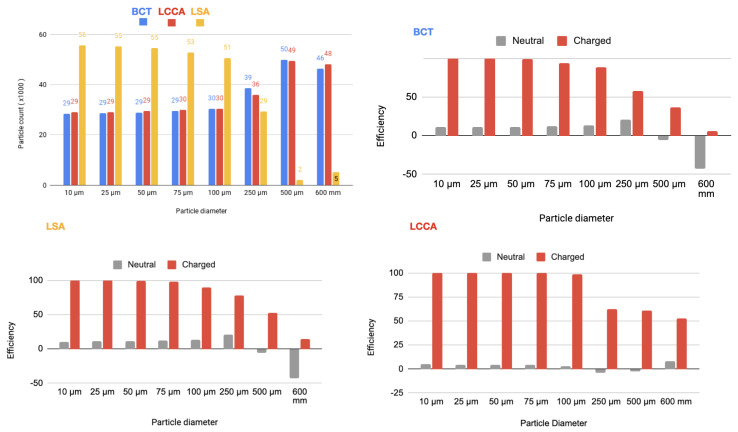
HP/TAVI—Field 4 (based on a device superficial charge of −100,000 [statC/m^2^]): Efficiency in each artery.

As it is described below, 64 simulations are carried out. In each of one, for each size of the particles, two electrical charge conditions were considered: neutral and electrically charged. The thrombus deflecting performance of the electrically charged device was evaluated with a flow rate waveform representative of a healthy patient (see section Blood flow model: computational fluid dynamics of the appendix provided in supplementary material of our previous work [22]). This waveform corresponds to a heart rate of 70 bpm and a cardiac output of $4286\;L\;\min^{-1}$. As a baseline, the simulation was first run without deploying the deflector, recording the number of particles of each type exiting each arterial outlet of the domain. Then, the device was deployed in all of the 3 aortic arch arteries simultaneously, repeating the process with particle filtering efficacy shown in the tables inserted in Figure 4 and represented in the histograms corresponding the statistical results of each scenario:

- Figure 5 Scenario Field 1 (based on a device superficial charge of −24 k statC/m^2^). This figure represents the global histograms en each artery showing the number of counted particles going through each of them (a) and histograms comparing neutral and charged scenarious in each artery, (b), (c) and (d).
- Figure 6 Scenario Field 2 (based on a device superficial charge of −48 k statC/m^2^). This figure represents the global histograms en each artery showing the number of counted particles going through each of them (a) and histograms comparing neutral and charged scenarious in each artery, (b), (c) and (d).
- Figure 7 Scenario Field 3 (based on a device superficial charge of −75 k statC/m^2^). This figure represents the global histograms en each artery showing the number of counted particles going through each of them (a) and histograms comparing neutral and charged scenarious in each artery, (b), (c) and (d).
- Figure 8 Scenario Field 4 (based on a device superficial charge of −100 k statC/m^2^). This figure represents the global histograms en each artery showing the number of counted particles going through each of them (a) and histograms comparing neutral and charged scenarious in each artery, (b), (c) and (d).

It is important to emphasize that the primary focus of this study is to prevent particles from passing through the LCCA artery. In all four presented fields, we observed a significant improvement in the filter device’s efficiency under the electric configuration. In the other arteries, we also observed a significant improvement in the filter diverter’s performance under the electrical scenario, with only a few exceptions related to larger particles and, only in some cases, always related with the LSA artery.

### 3.2. Atrial Fibrialtion Patient

#### Comparison Between 4 Intensities of Electric Field Repulsion and No-Charged Device

**Figure 9:**
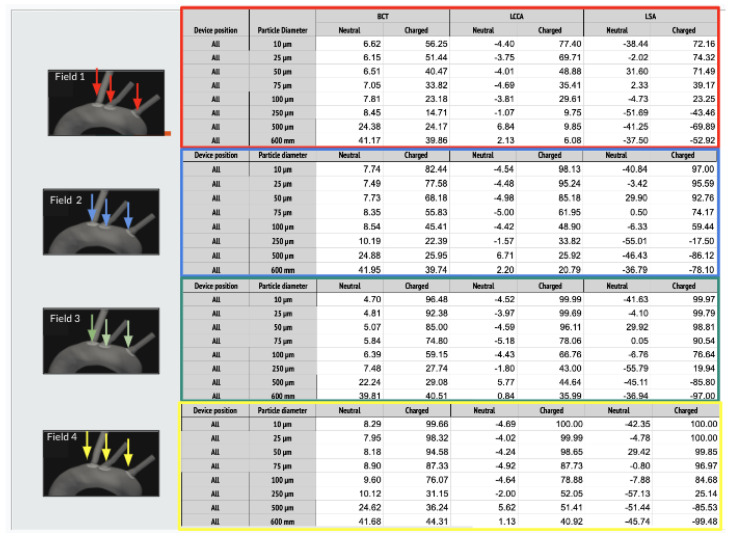
AF patient: Tables of efficiency defined in Equation (1) for all electrical fields (ranging from 1 to 4 different intensities) generated with the applied superficial charge: (−24,000, −48,000, −75,000, −100,000 [statC/m^2^]) on the three devices positioned at the bases of the arteries, illustrated with the arrows.

**Figure 10:**
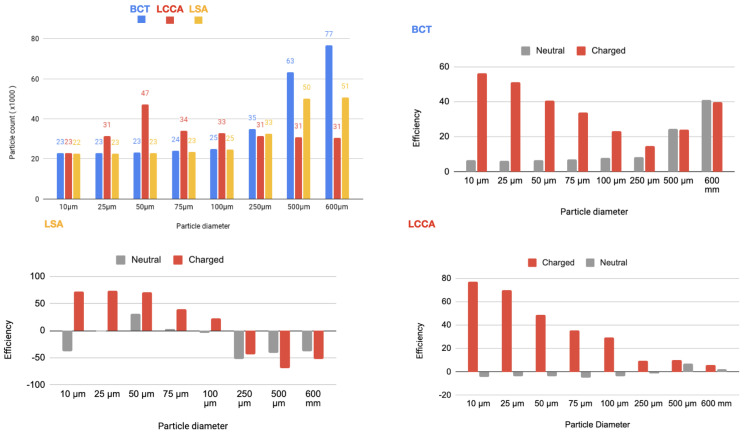
AF patient—Field 1 (based on a device superficial charge of −24,000 [statC/m^2^]): Efficiency in each artery.

**Figure 11:**
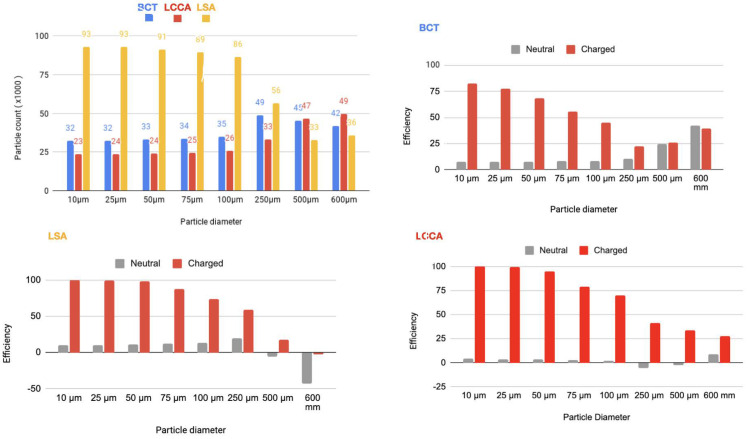
AF patient—Field 2 (based on a device superficial charge of −48,000 [statC/m^2^]): Efficiency in each artery.

**Figure 12:**
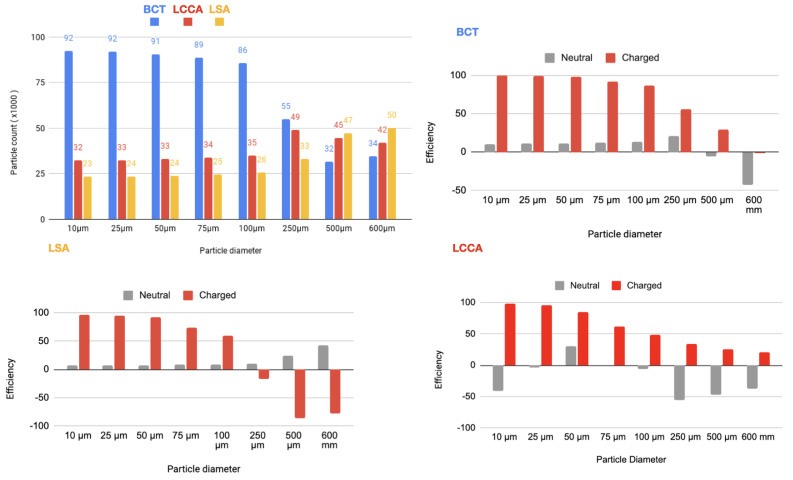
AF patient—Field 3(based on a device superficial charge of −75,000 [statC/m^2^]): Efficiency in each artery.

**Figure 13:**
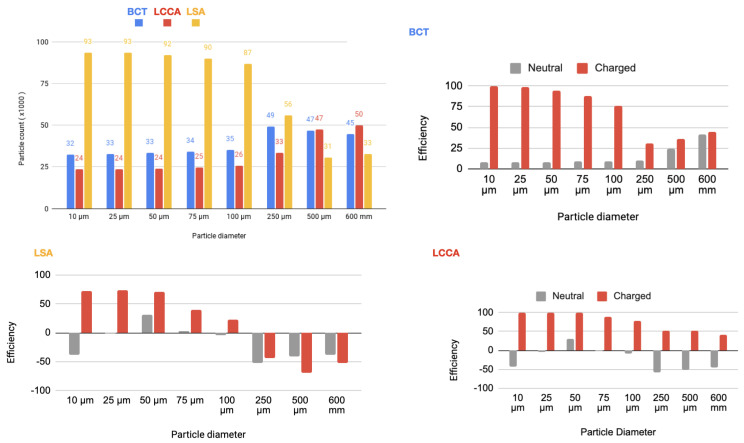
AF patient—Field 4 (based on a device superficial charge of −100,000 [statC/m^2^]): Efficiency in each artery.

In a second step, a flow rate waveform representative of an AF patient was imposed at the inlet. This waveform corresponds to a heart rate of 150 bpm and a cardiac output of $3429\;L\;\min^{-1}$, which corresponds to a 20% decrease with respect to the healthy patient [24]. As for the previous section, the device has been deployed in all three arteries simultaneously. The particle filtering efficacies are shown in tables inserted in Figure 9 and represented in the histograms corresponding the statistical results of each scenario:

- Figure 10 Scenario Field 1. This figure represents the global histograms en each artery showing the number of counted particles going through each of them (a) and histograms comparing neutral and charged scenarious in each artery, (b), (c) and (d).
- Figure 11 Scenario Field 2. This figure represents the global histograms en each artery showing the number of counted particles going through each of them (a) and histograms comparing neutral and charged scenarious in each artery, (b), (c) and (d).
- Figure 12 Scenario Field 3. This figure represents the global histograms en each artery showing the number of counted particles going through each of them (a) and histograms comparing neutral and charged scenarious in each artery, (b), (c) and (d).
- Figure 13 Scenario Field 4. This figure represents the global histograms en each artery showing the number of counted particles going through each of them (a) and histograms comparing neutral and charged scenarious in each artery, (b), (c) and (d).

As noted earlier, in all four presented fields, we observed a significant improvement in the filter device’s efficiency under the electric configuration. However, due to the altered fluid flow patterns in AF patients, the electrical forces have less impact on larger particle sizes in the LSA artery.

### 3.3. Discussion

In this work, the effectiveness of the filter/diverter stent is considered according to a study based on Computational Fluid and particle dynamics and as a continuation of a previous work by the same team [22], aimed at preventing stroke and SBIs, issues becoming more prevalent due to aging populations and increasing AF cases.

The device’s struts were given a negative electrostatic charge to repel thrombi, leveraging their natural electronegative properties. Combining computational fluid dynamics and electrostatics, the results showed the charged device efficiently filters particles of different sizes, notably the smallest ones, clearly outperforming the uncharged version.

#### 3.3.1. Main Conclusions

- Its action as a diverter, considering its effect of repelling microparticles (10 to 600 microns) due to the electronegative charge of the device, is demonstrated in the comparative tables of the different supra-aortic trunks (see Figure 4 and Figure 9. We explored microparticles’ sizes of 10, 25, 50, 75, 100, 250, 500 and 600 µm. Considering the charge/mass ratio, as the mass increases, the effectiveness decreases. We obtained more than 75% efficiency for the most dangerous ones, in the LCCA and the LSA.
- The second action of the device, as a filter, is demonstrated by the design characteristics (strut thickness of 0,75 or 750 microns and the distance between them of 750 microns combined with the convexity) thanks to the Coanda and other hydrodynamic effects. This allows a change in the flow waves and adds an antifouling washing effect due to the high velocity of aortic flow. According to the values obtained from the computational model, the so-called large cerebral vessel occlusion (LCVO), observed both in atrial fibrillation (AF) and TAVI, could theoretically be reduced in 100% of cases, offering elderly fibrillated patients the possibility of not needing anticoagulants by preventing stroke.
- Finally, we could infer an additional approach for anticoagulated AF patients who suffer a disabling stroke episode with the finding of cerebral micro-bleeds (MB) on MRI [8]. The therapeutic dilemma lies in the timing of resuming anticoagulant treatment, with the risk of exacerbating the hemorrhage or increasing the likelihood of a new embolic event. Perhaps a solution would be using the device and the total suppression of anticoagulation.

In summary, we consider that, theoretically, this work discusses the potential benefits of a Stent Filter/Diverter device in reducing the incidence of SBI and associated negative effects such as cognitive decline, depression, and dementia in patients with Atrial Fibrillation and those undergoing Transcatheter Aortic Valve Implantation (TAVI). The effectiveness is estimated to vary between 40% to 100%, depending on the size of the electronegative particles. It is important to look towards new paradigms, starting from the concept that “time is brain”. For this reason, new pharmacological technologies such as fibrinolytic and mechanical thrombectomy devices, have been developed. However, we must go a step further and consider how stroke episodes have decreased by addressing known risk factors (hypertension, diabetes, hypercholesterolemia, smoking, obesity, etc.). Nevertheless, the aging population and the increase in Atrial fibrillation in its different forms, atrial cardiomyopathy, and cerebral SVD, leading to cardioembolism, represent current and future challenges. We must achieve new developments or ideas, that reduce and try to avoid stroke, making the risk of use unnecessary as much as possible for the anticoagulants in the elderly population.

#### 3.3.2. Limitations

One of the limitations of the mathematical model used in this study is the assumption that clots are point-like particles in the fluid-particle interaction, as was discussed in detail in our previous work [22]. Particle tracking is performed by integrating Newton’s second law, meaning that mass affects the forces, but particle shape is not considered. Additionally, the fluid-particle coupling is one-way, meaning particles do not influence the fluid flow. These approximations are less accurate for particles significantly larger than the Kolmogorov scale of the flow, leading to increased errors with larger particles. Apart from limitations in the mathematical model, the physical parameters used in this study rely on scarce references in the literature and these parameters would require further validation through experimental data to enhance solidity. Examples of complex parameters include the blood’s permittivity, clot electric charge and size, and the charge of the graphene oxide coating on the device struts. Regarding the device’s geometrical model, a CAD artifact resulted in side struts with uneven thickness, thinning towards the ends. This created gaps of more than 1 mm in the device, which may have caused an artificial increase in the number of 500 µm particles entering the treated LSA in the healthy case. This issue will be assessed and corrected in future phases. This study serves as a proof of concept that demonstrates the potential of the proposed device and methodology, though given the novelty and inherent risks, extensive experimental validation is crucial moving forward.

In future studies, we will consider the feasibility of implanting a filter/diverter stent device in the three supra-aortic trunks, with and without electromagnetic loads in a virtual population. The study will follow parameters such as age (50–90 years), sex, hemodynamics, anatomical transformations of the aortic arch, size, angles, etc., using examples from CT angiography and MRI studies of real patients. This virtual population CFD study is a powerful weapon to assess the efficacy of the device, complementing clinical trials.

## Data Availability

The data presented in this study are available on request from the corresponding author. Some data that support the findings of this study are not publicly available due to privacy restrictions.
